# "The Hospital" Medical Book Supplement—No. XXXI

**Published:** 1910-07-16

**Authors:** 


### "THE HOSPITAL"
### MEDICAL BOOK SUPPLEMENT–No. XXXI

### CONTENTS.

| Medicine— | PAGE |
|---|---|
| The Pharmaceutical Pocket Book, 1910-1911 ... ... ... | 467 |
| Successful Cases of Puerperal Eclampsia ... ... ... ... | 467 |
| A Prescriber's Companion ... ... ... ... ... ... | 467 |
| Clinical and Pathological Papers from the Lakeside Hospital, Cleveland, U.S.A. ... ... ... ... ... ... ... ... | 467 |
| Memorias do Instituto Oswaldo Cruz ... ... ... ... | 467 |
| The Compendium of Medicine and Pharmacy ... ... ... | 463 |
| An Introduction to the Study of Hypnotism ... ... ... | 468 |
| Post-mortem Manual ... ... ... ... ... ... ... ... | 468 |
| Tuberculosis of the Nose and Throat ... ... ... ... | 463 |
| Sleeping Sickness ... ... ... ... ... ... ... ... | 468 |

| Miscellaneous— | PAGE |
|---|---|
| Ideal Health ... ... ... ... ... ... ... ... ... | 469 |
| London Pride and London Shame ... ... ... ... ... | 469 |
| Golden Rules of Obstetric Practice ... ... ... ... ... | 469 |
| The Athletic Life in its Relation to Degenerative Changes in the Cardio-Vascular System ... ... ... ... ... ... | 4(9 |
| Super-Organic Evolution ... ... ... ... ... ... ... | 469 |
| "The Swedish Exercise Treatment in Dealing with Injuries and Chronic Diseases" ... ... ... ... ... ... | 470 |
| Duties of the School Nurse ... ... ... ... ... ... | 470 |
| The Modern Scientific Treatment of Stammering and Stuttering ... ... ... ... ... ... ... ... ... | 470 |
| Missionaries and the Campaign against Malaria ... ... ... | 470 |
| The Action of Extracts of the Pituitary Body ... ... ... | 470 |

### MEDICINE.

THE PHARMACEUTICAL POCKET BOOK, 1910-1911. Edited by JOHN HUMPHREY. (London: The Pharmaceutical Press.)

THIS useful compendium is designed, as its title implies, for dispensers and chemists rather than for doctors. Nevertheless, since by stress of circumstance so many medical men are obliged to do their own dispensing, it is probable that to a considerable proportion of the profession it will prove extremely useful. There are, as a matter of fact, several sections of this extremely compact volume that are of great medical interest, even to those who have nothing to do with dispensing. The excellent tables of synonyms, wherein the real name of any drug and the variety of trade-mark or patented names under which it masquerades may be seen at a glance; the tables of dosage; the chapter on examination of urine; the sections on poisons, antidotes, the treatment of poisoning cases, and bacteriology: all these are valuable to every practitioner. Broadly speaking, however, those who prescribe only can obtain the information which they require on these subjects elsewhere; but those who also dispense will find the pages of Mr. Humphrey's book a great help in surmounting the numerous difficulties over which it is so easy to stumble in this distinctly intricate art. As a very minor point it may be noted that the information about scholarships on p. 370 has not been brought up to date.

SUCCESSFUL CASES OF PUERPERAL ECLAMPSIA. By ROBERT EMMET COUGHLIN, M.D., Brooklyn, N.Y. (Reprint from "American Medicine," March 1910. Pamphlet of 14 pages.)

AFTER discussing the Roman Catholic teaching, according to which it is never lawful to save the mother's life at the expense of the defenceless child, the author proceeds to give an account of seven cases of puerperal eclampsia treated by Dr. E. Cornwall and himself at the Norwegian Hospital. Four cases were primiparæ, three multiparæ. The ages of the patients were 20, 24, 30 (in two cases), 33 (in two cases), and 39 years respectively. In treatment no particular attention was paid to the fœtus, the idea being to save the mother. All the mothers recovered and left the hospital perfectly well. Three infants were born alive, and four born dead. The treatment pursued was first the employment of certain drugs to ameliorate symptoms, and the use of the hot-pack. The main idea however, was to terminate pregnancy, no matter what the period of utero-gestation. Labour was induced in five cases by the catheter and forcible dilatation, forceps being applied in two of them. In one case delivery occurred spontaneously shortly after reaching hospital, and in another delivery occurred before admission. The author has faith in veratrum viride in the treatment of the convulsions, and in atropine sulphate in desperate cases where pulmonary œdema is imminent. After delivery, treatment consists in proper dieting, careful nursing, and the application of the hot-pack to the kidney region.

A PRESCRIBER'S COMPANION. By THOMAS D. SAVILL, M.D. Lond. Fourth edition. Revised by the author, assisted by CHARLES F. HARFORD, M.A., M.D.Cantab. (London: Henry J. Glaisher. 1910. Price 1s. net.)

THE lamented death of the author of this little volume lends an additional interest to the appearance of this, the fourth edition. Those who have used a former edition of this useful work will need no introduction to the present one, which worthily maintains the standard of excellence aimed at. Several new prescriptions have been added, and a number of the old ones omitted. In the second part, which deals largely with treatment, some of the sections have been entirely rewritten to bring them up to the present-day standard of knowledge. The only criticism that we have to offer is that in our opinion space should have been given for the addition of the practitioner's own favourite formulæ, which could have been provided by interleaving the first part. The resources of the Pharmacopœia can hardly be said to have been exhausted by the provision of 138 prescriptions!

CLINICAL AND PATHOLOGICAL PAPERS FROM THE LAKESIDE HOSPITAL, CLEVELAND, U.S.A. (1910. Pp. 365.)

THIS, the fourth, series of reports issued by the hospital contains 22 papers by the members of the medical staff, who have adopted the method of publishing from time to time in a single volume the more important papers from the different services of the hospital which have appeared in the various medical journals. This method leaves each author free to publish the results of his work in the medical journal of his choice, while by the collection of reprints of these papers in an annual volume all the purposes of a special hospital publication are subserved. It is impossible here to discuss the papers contributed, but among others there are articles on gastric ulcer, intestinal hæmorrhage in typhoid fever, perineal herpes after pneumonia, vaccine therapy, foot deformities, the mechanism of hæmolysis, colloid glands and their relation to iodine, and on parapsoriasis.

MEMORIAS DO INSTITUTO OSWALDO CRUZ. (Rio de Janeiro: Manguinhos. Tomo I. Faciculo II. Dezembro 1909. Pp. 218.)

THIS well printed and beautifully illustrated report of work carried on at the Institute of Oswald Cruz in Rio de Janeiro contains eight articles on various subjects connected with bacteriology and parasitology. Each article is printed in two languages—Portuguese and another. The second language in most cases is German, but one article by Dr. Gomes de Faria, on "Echinostomum Crotophagar. n. sp., a new parasite of the blue anú *Crotophaga major*)," is translated into English. We must congratulate Mr. Castro Silva on the beauty of his drawings, of which there are many in the text, and the unnamed printer on the manner in which they have been reproduced.

The Compendium of Medicine and Pharmacy. By C. J. S. Thompson. (London : John Bale, Sons, and Danielsson. 1910. Third edition. Price 5s. net.)

This little work is a veritable *multum in parvo.* While somewhat too bulky for pocket use, space can easily be found for it on the practitioner's table, to whom it will serve as a handy book of reference, for there are few subjects connected with drugs with which it does not deal to some extent. A synopsis of recent and unofficial remedies and formulæ, the usual test tables, as well as suggestions for invalid diets are all to be met with in its pages. A short account is given of the drugs and preparations met with in the United States, French, German, and British Pharmacopœias, together with the Indian and Colonial addendum to this last. The book should be particularly useful to those who compound their own medicines.

An Introduction to the Study of Hypnotism. By H. E. Wingfield, M.D. (London : Baillière, Tindall and Cox. 1910. Pp. 175. Price 5s. net.)

Treatment by suggestion is developing an increased sphere of usefulness in medicine now that its methods are becoming better understood. Increased knowledge is proving beyond doubt that as a therapeutic measure hypnosis and suggestion should have a much higher place than they have had in the past, for, although there is the great danger of their denegrating into quackery when they are employed by unscrupulous persons, rightly used by practitioners of repute they are sometimes extremely valuable. The book before us contains nothing particularly new, but it affords a good summary of the phenomena of hypnosis. Unfortunately, of the seven chapters of the book one only is devoted to treatment by suggestion. Medical practitioners need a good concise work which might be described as an elaboration of chapter vi.

Post-mortem Manual. By Charles R. Box, M.D., F.R.C.P. (London : J. and A. Churchill. 1910. Pp. 335. 8vo. Price 6s. net.)

This manual is intended essentially for the post-mortem room, and it does not enter into details of microscopical examination or of bacteriological identification. It gives precise directions as to how a post-mortem examination should be made, with a description of the instruments required and of the points to be noticed both externally and internally. There is an immense amount of information compressed into its small bulk, and it will be found useful not only to practitioners who are called upon to make post-mortem examinations after they have left the hospital, but also to students who wish to learn the different lesions that may be presented by any of the various tissues or viscera. If there is any general criticism that one would make of the work it is that it does not sufficiently emphasise the difference between those changes which are common and those which are rare, but otherwise the information given is, upon the whole, sound. There are points with which we disagree—for instance, the relatively prominent position given to tuberculosis as a cause of pericarditis; it is extremely rare to find tuberculous pericarditis, notwithstanding the statements in more than one text-book to the contrary. The characteristic flea-bitten appearance of the kidneys of malignant endocarditis is not insisted on. The weight of the thymus gland is given as "7 to 14 grams or more," whereas it is not at all uncommon to find it weighing 30, 40, or 50 grams without it necessarily being diseased. We are pleased to see that the author speaks of status lymphaticus as "being looked upon by some authorities as producing glottic spasm or sudden death from syncope"; we agree with what Dr. Box seems to imply, namely, that the so-called status lymphaticus is a very doubtful entity. It is a question whether this status lymphaticus is not the normal condition in healthy children who are suddenly killed whether by accident or by anæsthetics. Figure 11 on page 105 would seem to imply that Dr. Box considers two persons, that is to say, two pairs of hands, are needed for the proper examination of the bowel, but it is really quite easy for a person to examine the intestines without assistance. Notwithstanding little points of this kind we congratulate Dr. Box upon his manual, and we recommend it as a work upon a subject that is both useful and of a convenient size.

Tuberculosis of the Nose and Throat. By L. B. Lockard, M.D. (St. Louis : C. V. Mosby Publishing Co. 1909.

Text-books on diseases of the nose and throat have increased to such an unnecessary number that it is refreshing to find a laryngologist writing something more approaching a monograph, and a very good one into the bargain. While well cognisant of the vast recent literature, the author has taken standard works to guide him to original sources, and so produced a good historical account of the growth of our knowledge of these subjects. Moreover, he has a good array of personal cases with which to cap previous statistics. He follows the recent tendency to use formalin for laryngeal ulcers instead of lactic acid, and does not overdo active treatment, although his moderation might be further increased by seeing the results of letting a series of cases severely alone, except for vocal rest and general treatment. Galvano-puncture he has apparently not very much experience of, and the slight value he attaches to orthoform and anæsthesin will probably surprise British practitioners. The growing subject of tuberculosis of the tonsils is well treated. No opinion is expressed as to whether or not direct connection exists between the bronchial glands and the cervical lymphatics. Here possibly acquaintance with the studies of Most, reinforcing those of Beitzke and of Wood, might have decided the author. A good deal too is missing from the section on non-tuberculous nasal abnormalities in consumptives. But this is an excellent book, and the illustrations are remarkable—we have never seen such a good collection of pictures of the mirror-image in laryngeal tubercle. There should have been a separate bibliography; nowadays it is as necessary as an index.

Sleeping Sickness : How to avoid infection; with an account of Glossina Palpalis, and illustrations of this and other biting flies. For the use of Travellers and Residents in Tropical Africa. Issued under the direction of the Honorary Managing Committee. (London : Sleeping Sickness Bureau. 1910. Pamphlet of 15 pages, with eight illustrations.)

A concise account of the main points of difference between the various African biting-flies, with special reference to the methods of distinguishing Glossina Palpalis from the others. An account is given of the life-history of the fly, its method of reproduction, and its habits. Then follows a description of sleeping sickness, with advice as to its prevention. The little book is well written and clearly printed, the illustrations are excellent, and most important of all, the subject-matter is compiled by recognised authorities on the subject with which it deals.

### MISCELLANEOUS.

Ideal Health. By "M.D." (Bristol: J. Wright and Sons. Price 1s.)

From a medical point of view this book is useless, but as an aid to various gymnastic exercises it may be of some service. Many and varied are the exercises so described, both for dumb-bells and for the "spring chest expander"—an article that is advertised at the end of the book, and to sell which the book seems to have been written. Only in the capacity of a drill instructor is it to be recommended.

London Pride and London Shame. By L. Cope Cornford. (London: S. King and Sons. Orchard House, Westminster. 6s. net.)

Centuries ago that much maligned man who now sleeps quietly under the linden-trees in the little Salzburg churchyard, expressed himself clearly, a trifle vehemently, on the subject of thoroughness. Not merely his bitterness, his Berserk rage at the crass stupidity of his contemporaries, made Paracelsus keen to see the faults of his time; there was added to this draught of the water of Amara a dash of his own opium, that dulled for the moment the ambition that ruined him and gave him a chance to philosophise. "Drink not of a standing pool, but gulp at a river, and gulp deep." "If thou seest ruin and shame ask thyself how can it be prevented, and set thyself to prevent it. I know, I that have suffered it, what the world will pay. Those that buzz and flutter sit in high places and eat venison larded and drink Dalmatian wine; those that work and use their brains huddle in hovels with the cold draught blowing over them, and sup upon bread broth." Perhaps Mr. Cornford knows his Paracelsus and the reward that the world weighs out to reformers. He has learnt his London—part of it at least—and seen how its people, its poor especially, exist. His experiences have supplied him with "copy," and he has set himself to write them down, with an extra dash of sentiment, an extra supply of adjectives, and a sackful of trite sayings that can be sprinkled through the whole to spice it. Our chief count against him is that he has not gulped deeply, and that he has not asked himself, How may I help in reforming all this? At least he has given us no ground for believing, in spite of his elaborate apology in the preface, that he has thought about it at all. We find these short essays superficial and weak. To take the three that are of special interest to medical men—famine, medical, and surgical—they display a wonderful ignorance of detail, the sort of thing one looks for from Mr. Begbie or Mr. Bart Kennedy, not from one who is a Paracelsian, and goes to the root of things. The problem of poverty in London—and in all great cities, is not a sentimental problem, not a question that can be effectually dealt with by armchair philosophers, and we are much afraid that Mr. Cornford does not impress his reader with his ability to deal with it. His little essays are readable enough, but they do not take one deeply into the questions with which they deal. Some of them make far too much capital out of that shoddy clap-trap which nowadays passes for imperial sentiment, but the author is probably intensely sincere and will feel hurt when we tell him that a colonial reads his most fervent periods without a thrill, and sometimes with intense amusement. So, too, we imagine the average surgeon will read the essay entitled Surgical; it recalls a column of nonsense that appeared some years ago in the *Daily Mail* on a somewhat similar subject. The other essays are not without interest, for example, those dealing with the training-ship *Mercury* and the Guild of the Brave Poor Things, and are instructive as well. As an armchair reading-book this work has its merits, and the practitioner who has an hour to spare will find its perusal instructive and interesting.

Golden Rules of Obstetric Practice. By W. E. Fothergill, M.A., B.Sc., M.D. ("Golden Rule" Series. No. III. (Bristol: John Wright and Sons, Ltd. 1910. Price 1s.)

The continued popularity of this little compendium of obstetric maxims is amply evidenced by the appearance of this, the sixth edition. The distinguished obstetrician who is responsible for its appearance has carefully revised the book throughout and brought it up to date. Framed for the use of beginners in practice, and accordingly based on broad and well-tried principles, these rules will be found useful to many who have left their student days behind.

The Athletic Life in its Relation to Degenerative Changes in the Cardio-Vascular System. By Robert E. Coughlin, M.D. (New York: William Wood and Co. 1910. Pamphlet of 18 pages.)

After rapidly reviewing the opinions of authorities of all nationalities for and against athletic exercises, the author concludes that on the whole the evidence at hand seems to be against school-athletic contests unless adequate precautions be taken to prevent overstrain. He wholeheartedly condemns Marathon contests both for boys and men, and declares that the death of the first Marathon runner should have been an object-lesson for all time. He believes that in athletes the foundations of an intemperate life are often laid by the cry of the over-developed heart-muscle for stimulation. As a rule men over forty do not take exercise sufficient for the maintenance of metabolism. Active exercise should be kept up beyond middle life, especially in those who have led an athletic life in youth, for there is a distinct relation between want of muscle-tone after middle age and the development of changes in the cardio-vascular system. The best exercise is brisk walking in the open air.

Super-organic Evolution. By Dr. Ernique Lluria. Translated by Rachel Challice and D. H. Lambert. (London: Williams and Norgate, Covent Garden. Price 8s. 6d. net.)

Dr. Lluria's work is well known to students of evolution and social questions, for the author has won recognition as an authority whose opinions are worth consideration. Nevertheless these opinions will not be generally accepted by the mass of the medical profession who have not made the study of evolution, ordinary or super-organic, a part of their intellectual curriculum. For many the questions touched upon will be too deep, too profound, and too much of the category in dealing with which the question "To whom the profit?" invariably arises. It needs a fairly close acquaintance with modern speculative philosophy of the kind that biologists admire to follow Dr. Lluria, clear and incisive though his manner of exposition may be. Modern evolutionists remind one very forcibly of the second class of philosophers in the Doctor of Literature's study, so graphically described by Dekker: "The gentlemen who were much more stupid than those who frankly declared that they knew nothing, because they were fools enough to pretend that they did know something." These gentlemen nowadays do not found systems of esoteric philosophy, they write original treatises of heredity in which they out-Weismann Weismann. Fortunately, Dr. Lluria is not of their class, his speculations are not dogmatic, they are merely suggestive, and if the suggestions sometimes take our breath away it is due to the importance and complexity of the subject, not to the involvement and artificial intricacy of the author's style or to the vagueness of his definitions. The work of translation has been admirably and idiomatically done. We can only wonder how it comes that the translators have not mastered their subject sufficiently to write the correct names of foreign authorities, who in this volume masquerade under guises which make them unrecognisable. Who is *de Wries*, for instance? The spelling is even phonetically wrong.

The general practitioner who endeavours to read outside his merely professional lines, to touch something that is stimulating, and at the same time suggestive, cannot do better than peruse this book. Dr. Ramon y Cajal contributes an interesting preface, in which the subject of the book is approached from a standpoint entirely different from that of the author, though the conclusions, so far as the physiologist allows himself to conclude, are more or less identical. Dr. Lluria's philosophic standpoint is somewhat difficult to define. He is not a Spencerian nor a Positivist, and Socialists will not care to hail him as a comrade in thought. We leave the reader of his very interesting and original work to arrive at a decision with regard to his views—an occupation which will be fraught with much enjoyment and mental recreation. As an introduction to sociological questions considered in the light of modern science, and chiefly from the point of view of the biologist, the book will well repay the reading.

"The Swedish Exercise Treatment in Dealing with Injuries and Chronic Diseases." By Otto Leonard Holst, M.R.C.S. Eng., L.R.C.P. Lond., Graduate of the University of Lund, Sweden. (Eastbourne: Gowland Bros., Printers. 1910. Price 6d. net.)

The great principle on which Swedish exercise treatment is based is that of acting on the circulation, where faulty, and of regulating the flow of blood through all the organs. By such regulation much improvement, if not absolute cure, can be effected, especially in chronic disorders. After allowing for the natural enthusiasm of one who has been closely connected with the country from which these exercises derive their name, we are in the main inclined to agree with most of the author's statements, especially as to the early mobilisation and massage of fractures and dislocations, a form of treatment with which the name of Sir William Bennett is intimately associated in this country. In the short space at his disposal Dr. Holst has not attempted to do more than outline the treatment, but has contented himself with describing the conditions in which its application is likely to be of benefit.

Duties of the School Nurse. By S. C. McCall Knipe. (London: The Scientific Press, Ltd. 1910. Pp. 31. Price 1s. net.)

After rapidly surveying the chief qualifications and duties required of a school nurse, the author gives some valuable hints as to the examination of heads of the children, the treatment of minor ailments, the interviewing of mothers of dirty children, and the weekly visits to the schools. She explains the objects of medical inspection, and discusses the part played by the nurse during the doctor's inspection. The final chapter is devoted to the prospects of school nursing as a career. The work should prove useful to those who wish for information on the subject with which it deals. We found the print rather trying to read, on account of its smallness.

The Modern Scientific Treatment of Stammering and Stuttering. By J. Herbert Miall. (London: Grayson and Co. 1910. Price 3d.)

Having cured himself by the methods he advocates, the author herein sets out his views as to the predominant factor in the causation of these distressing conditions. He believes that unbalanced mental concentration in syllabication, the outcome of an erroneous method of teaching children to read, is the chief cause of stammering. This he proposes to cure by a suitable application of his own method of elocution in which he makes use of what he terms "vocal keys," and which he finds infallible when accompanied by rational breathing and articulatory exercises. As to what the precise details of this elocutionary cure are, the pamphlet leaves us in ignorance, but under the heading "The object of this treatise" the author expresses his willingness to give any assistance possible to those interested, upon application to himself.

Missionaries and the Campaign against Malaria. An Address by Ronald Ross, C.B., F.R.S., D.Sc., LL.D., F.R.C.S., Major I.M.S. (retired), Nobel Laureate, Professor of Tropical Medicine in the University of Liverpool. Prepared for the Commemoration Day Proceedings of Livingstone College, June 11, 1910. (Pamphlet of 14 pages. Price 2d.)

The author, one of the greatest living authorities on the subject of Malaria, addresses an appeal to the representatives of the missionary societies, inviting them to join in a great campaign against malaria and other tropical diseases. He briefly outlines methods of prevention and cure, and advises as to when it is best to employ preventive measures. He recommends that every missionary should receive some elementary instruction in tropical hygiene, and, while not wishing to puff his own work, strongly advises such missionary to commence by buying some standard book such as his own "Prevention of Malaria," in which the subject is dealt with by twenty leading authorities.

The Action of Extracts of the Pituitary Body. By H. H. Dale, M.A., M.D. Reprinted from the "Bio-Chemical Journal," Vol. IV., No 9. (From the Wellcome Physiological Research Laboratories, Herne Hill, London, S.E. 1909.)

The author has collected in this pamphlet a number of observations made at different times and in different connections with regard to the action of pituitary extracts, from which he is led to believe that, while the action of these extracts have several points of similarity with that of thyroid extracts, such correspondence as exists is wholly superficial and illusory. In most experiments the author made use of a 5 per cent. decoction of the fresh posterior lobes of ox pituitaries, dissected clean from the rest of the gland and from dura-mater, weighed in the moist condition, pounded with sand, and boiled with water faintly acidulated with acetic acid to produce coagulation. The extract filtered from coagulum is a clear, colourless fluid giving a faint biuret reaction. He concludes as the result of his experiments that the action of extracts of the posterior lobe of the pituitary body is a direct stimulation of involuntary muscle, without any relation to innervation. The action is most nearly allied to that of the digitalis series, but the effect on the heart is in this case slight, and that on plain muscle intense. The active principle is excreted in the urine. No true immunisation can be produced by repeated injections of the extract. The evidence advanced in proof of the existence of separate pressor and diuretic principles is inadequate.

